# Serum exosomal tsRNA biomarkers: A novel strategy for identifying lupus nephritis

**DOI:** 10.1002/ctm2.1677

**Published:** 2024-05-17

**Authors:** Ping Yang, Yifan Sun, Chenlan Wang, Zhibo Li, Yiyuan Han, Jianming Gong, Adeel Khan, Jin Wang, Yanbo Wang, Fangfang Jin, Zhiyang Li

**Affiliations:** ^1^ Department of Clinical Laboratory The Affiliated Drum Tower Hospital of Nanjing University Medical School Nanjing China; ^2^ State Key Laboratory of Pharmaceutical Biotechnology Department of Physiology Jiangsu Engineering Research Center for MicroRNA Biology and Biotechnology School of Life Sciences NJU Advanced Institute of Life Sciences Nanjing University Nanjing China; ^3^ Department of Laboratory Medicine Nanjing Drum Tower Hospital Clinical College of Jiangsu University Nanjing China; ^4^ Jiangsu Collaborative Innovation Center of Traditional Chinese Medicine Prevention and Treatment of Tumor, School of Medicine & Holistic Integrative Medicine Nanjing University of Chinese Medicine Nanjing China; ^5^ Department of Biotechnology University of Science and Technology Bannu Pakistan

Dear Editor,

For effective treatment and control of systemic lupus erythematosus (SLE), it is pertinent to be able to precisely identify and predict lupus nephritis (LN).^1^ The current gold standard for this is renal biopsy, but this is invasive, making it less attractive for dynamic monitoring of disease progression.^2^, ^3^ tRNA‐derived small non‐coding RNAs (tsRNAs), as a novel RNA biomarker, intricately involved in regulating various stages of gene expression, from transcription, translation, RNA processing, to maturation, are implicated in key cellular processes, including self‐renewal, differentiation, proliferation and onset of pathological conditions such as immune system disorders and cancer.^4^, ^5^, ^6^, ^7^ Exosomes, which typically range from 30 to 100 nm, derived from cells, serve as pivotal mediators for intercellular communication and also act as crucial carriers for circulating tsRNA.^8^ Here, we anticipated and demonstrated that serum exosome‐encapsulated tsRNAs can serve as biomarkers for executing LN diagnosis noninvasively.

The study recruited participants (HC, *n* = 80; SLE (LN‒), *n* = 122; LN, *n* = 131) from Nanjing Drum Tower Hospital between September 2020 and June 2022, in accordance with the 1997 SLE classification criteria^9^ set by the Rheumatology Society (Table 1). Ethical approval was obtained from the Ethics Committee of Nanjing Drum Tower Hospital, with the approval identification number 2020‐327‐01. An exosome isolation kit (from serum) (Thermo Fisher Scientific Inc.) was initially used for exosome separation from 100 µL of serum. The exosomes were then characterised by transmission electron microscopy, nanoparticle tracking analysis and Western blotting (Figure S1). RNA from the serum exosomes was extracted using Trizol and treated with an rtStart tRF and tiRNA pre‐treatment kit (Arraystar Inc.) prior to sequencing. The volcano plot showed that 88 tsRNAs were increased and 66 tsRNAs were decreased based on the criteria of fold change greater than 2 and a *p*‐value less than 0.01 (Figure 1A). The species and occupancies of the nine types of tsRNAs had no obvious differences between SLE (LN‒) and LN groups (Figure 1B), while the fragment lengths of tsRNAs were mainly enriched in the 20−22 nt and 31−32 nt intervals (Figure 1D). The Venn distribution indicated that 195 tsRNAs existed simultaneously in both groups, 84 tsRNAs were unique to the LN group and 64 tsRNAs were unique to the SLE (LN‒) group (Figure 1C). Ultimately, we plotted the column chart of the top 10 high/low‐expression tsRNAs in the two groups (Figure 1E) and preliminarily verified the actual expression of the 10 high‐expression tsRNAs in the LN group relative to SLE (LN‒) using RT‐qPCR (Figure 1F).

**TABLE 1:** Clinical and immunological characteristics of the systemic lupus erythematosus (SLE) participants.

	Training		Verification		
Index	SLE (LN‒)	LN	SLE (LN‒)	LN	HC
Cases	17	23	105	108	80
Gender (male/female)	1/16	1/22	7/98	5/103	17/63
Age (year)	42.71 ± 17.33	47.13 ± 15.80	44.30 ± 16.33	40.33 ± 14.69	35.96 ± 9.88
VD (ng/mL)	N/A	N/A	17.24 ± 10.82	18.75 ± 13.34	N/A
SLEDAI‐2K	6.75 ± 4.46	11.35 ± 4.44	7.84 ± 8.47	10.07 ± 9.39	N/A
24h‐UP (mg/L)	226.98 ± 122.49	3566.93 ± 3433.64	157.90 ± 138.20	2646.48 ± 3139.91	N/A
ESR (mm/h)	N/A	N/A	45.82 ± 34.89	44.64 ± 29.39	N/A
eGFR	140.05 ± 41.51	86.75 ± 49.29	131.40 ± 54.09	96.34 ± 67.73	124.63 ± 23.13
CRP (mg/L)	4.98 ± 4.26	11.89 ± 16.95	16.70 ± 34.95	15.07 ± 28.91	N/A
C3 (g/L)	.62 ±.29	.60 ±.30	.78 ±.29	.80 ±.37	N/A
C4 (g/L)	.09 ±.06	.11 ±.09	.12 ±.07	.18 ±.13	N/A
Anti‐β2‐GPI (RU/mL)	45.45 ± 75.81	2.96 ± 3.62	20.61 ± 49.27	7.92 ± 9.87	N/A
Anti‐dsDNA‐M (IU/L)	451.99 ± 490.31	313.83 ± 375.59	224.91 ± 304.00	302.60 ± 364.60	N/A
ANA (+) (percentage)	N/A	N/A	57 (54.29%)	67 (62.04%)	N/A
Anti‐dsDNA (+) (percentage)	3 (17.65%)	6 (26.09%)	8 (7.62%)	17 (15.74%)	N/A
ANuA (+) (percentage)	6 (35.29%)	8 (34.78%)	15 (14.29%)	30 (27.78%)	N/A
RNP (+) (percentage)	2 (11.76%)	5 (21.74%)	17 (16.19%)	22 (20.37%)	N/A
Anuc (+) (percentage)	1 (5.88%)	10 (43.48%)	28 (26.67%)	27 (25.00%)	N/A
ASMA (+) (percentage)	1 (5.88%)	5 (21.74%)	17 (16.19%)	14 (12.96%)	N/A

Abbreviations: 24h‐UP, 24‐h urine protein; ANA, anti‐nuclear antibody; anti‐dsDNA, anti‐double‐stranded DNA antibody; anti‐β2‐GP I, anti‐beta 2 glycoprotein 1 antibody; ANuA, anti‐nucleosome antibody; ASMA, anti‐Smith antibody; C3, complement C3; C4, complement C4; CRP, C‐reactive protein; eGFR, estimated glomerular filtration rate; ESR, erythrocyte sedimentation rate; LN, lupus nephritis; N/A, not available; RNP, anti‐ribosomal P protein; SLEDAI‐2K, systemic lupus erythematosus disease activity index 2000; VD, vitamin D.

**FIGURE 1:**
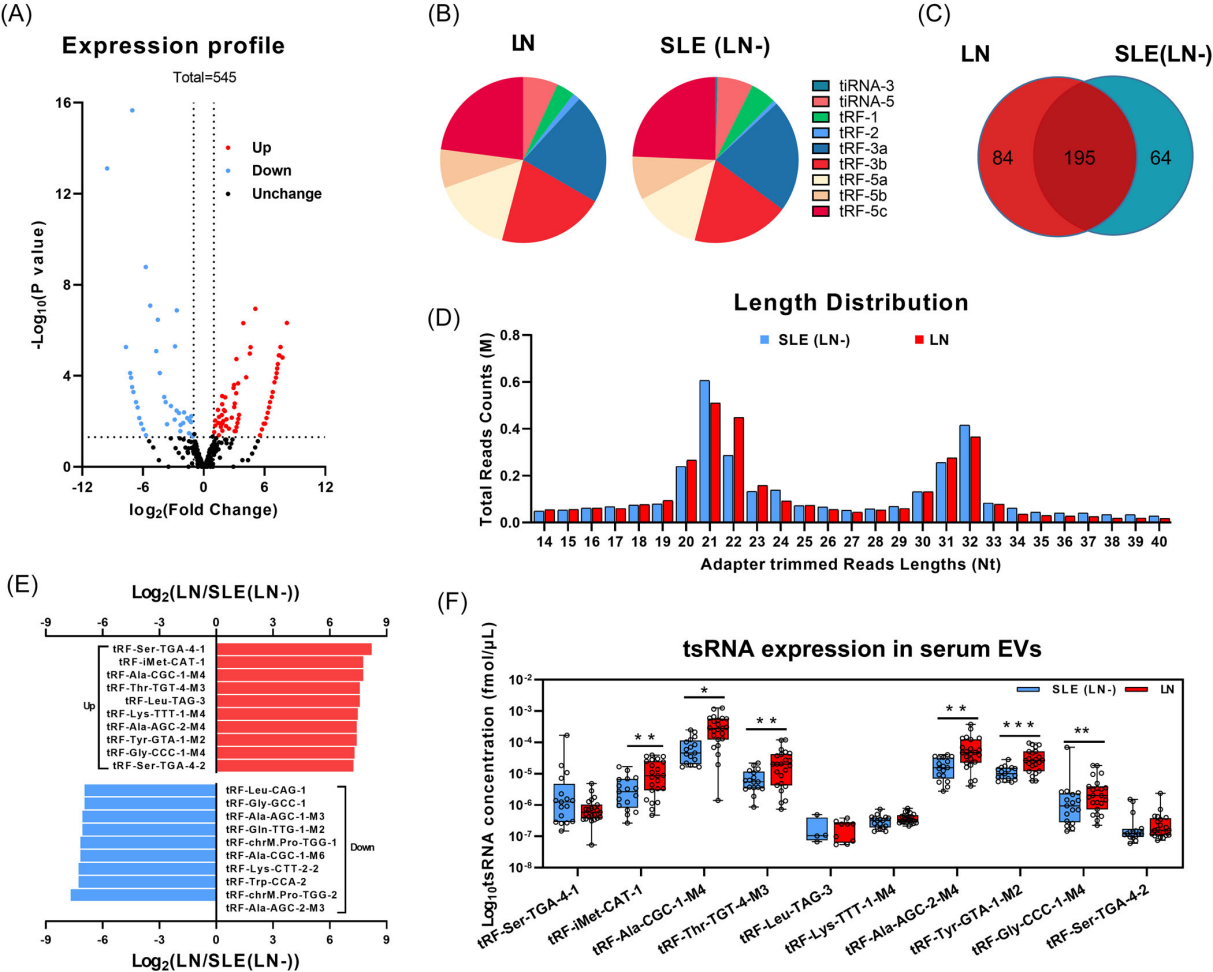
Development and screening of markers for exosome‐encapsulated tsRNAsin lupus nephritis (LN). (A) Volcano plot of differential expression of tRNA‐derived small non‐coding RNAs (tsRNAs) between systemic lupus erythematosus (SLE) (LN‒) and LN (threshold setting: the fold change > 2 and the *p*‐value <0.01). (B) Species distribution of tsRNAs in SLE (LN‒) and LN. (C) Venn distribution of tsRNAs. (D) Length distribution of tsRNAs in SLE (LN‒) and LN. (E) Expression heatmap of top 10 high/low‐expression tsRNAs in LN group compared to SLE (LN‒) group. (F) Clinical sample validation of 10 candidate tsRNAs between SLE (LN‒) and LN. A non‐parametric *U*‐test was used to analyse the differences between the two groups (^*^ *p* < .05, ^**^ *p* < .01, ^***^ *p *< .001).

Furthermore, tRF‐iMet‐CAT‐1, tRF‐Ala‐AGC‐2‐M4 and tRF‐Tyr‐GTA‐1‐M2 showed eminently high expression in the SLE (LN‒) group than in the HC group (Figure 2A,C,D). In contrast, tRF‐Thr‐TGT‐4‐M3 and tRF‐Tyr‐GTA‐1‐M2 were significantly higher expressed in the LN group than in the SLE (LN‒) group (Figure 2B,D). Even so, tRF‐Gly‐CCC‐1‐M4 showed no eminent expression differences between the two groups (Figure 2E). To evaluate the diagnostic value of the screened tsRNAs, we generated subject working receiver operating characteristic (ROC) curve and developed a random forest diagnostic model. Among the tested tsRNAs, tRF‐Tyr‐GTA‐1‐M2 exhibited the highest diagnostic efficacy in distinguishing HC from SLE (LN‒) with an area under the ROC curve (AUC) of 0.7663 (Figure 2F). tRF‐iMet‐CAT‐1, tRF‐Thr‐TGT‐4‐M3, tRF‐Ala‐AGC‐2‐M4 and tRF‐Tyr‐GTA‐1‐M2 showed high diagnostic values for the diagnosis of LN from HC, with AUCs over 0.86 (Figure 2G). tRF‐Thr‐TGT‐4‐M3 and tRF‐Tyr‐GTA‐1‐M2 had diagnostic values for the diagnosis of LN from SLE (LN‒), with AUCs of 0.6589 and 0.6499, respectively (Figure 2H). Subsequently, the correlation heatmap of five tsRNAs and 18 clinical indicators showed that tRF‐Tyr‐GTA‐1‐M2 had significant positive correlations with the SLE disease activity index score, 24‐h urine protein, C‐reactive protein and erythrocyte sedimentation rate (Figure 2I). Furthermore, according to the random forest algorithm, estimated glomerular filtration rate (eGFR) exhibited the highest mean decrease Gini in all clinical index (Figure 2J), with joint AUC 0.88 (Figure 2K); tRF‐Tyr‐GTA‐1‐M2 and tRF‐Thr‐TGT‐4‐M3 exhibited the highest mean decrease Gini in five tsRNAs (Figure 2L), with joint AUC 0.879 (Figure 2M). Finally, a machine learning modeling analysis was conducted by integrating four tsRNAs with six clinical indexes. It was observed that eGFR, tRF‐Thr‐GTA‐4‐M3 and tRF‐Tyr‐GTA‐1‐M2 exhibited the top mean decrease Gini (Figure 2N). Combined the top 10 diagnostic markers together, the AUC for the diagnosis of SLE (LN‒) and LN reached 0.919 (Figure 2O).

**FIGURE 2:**
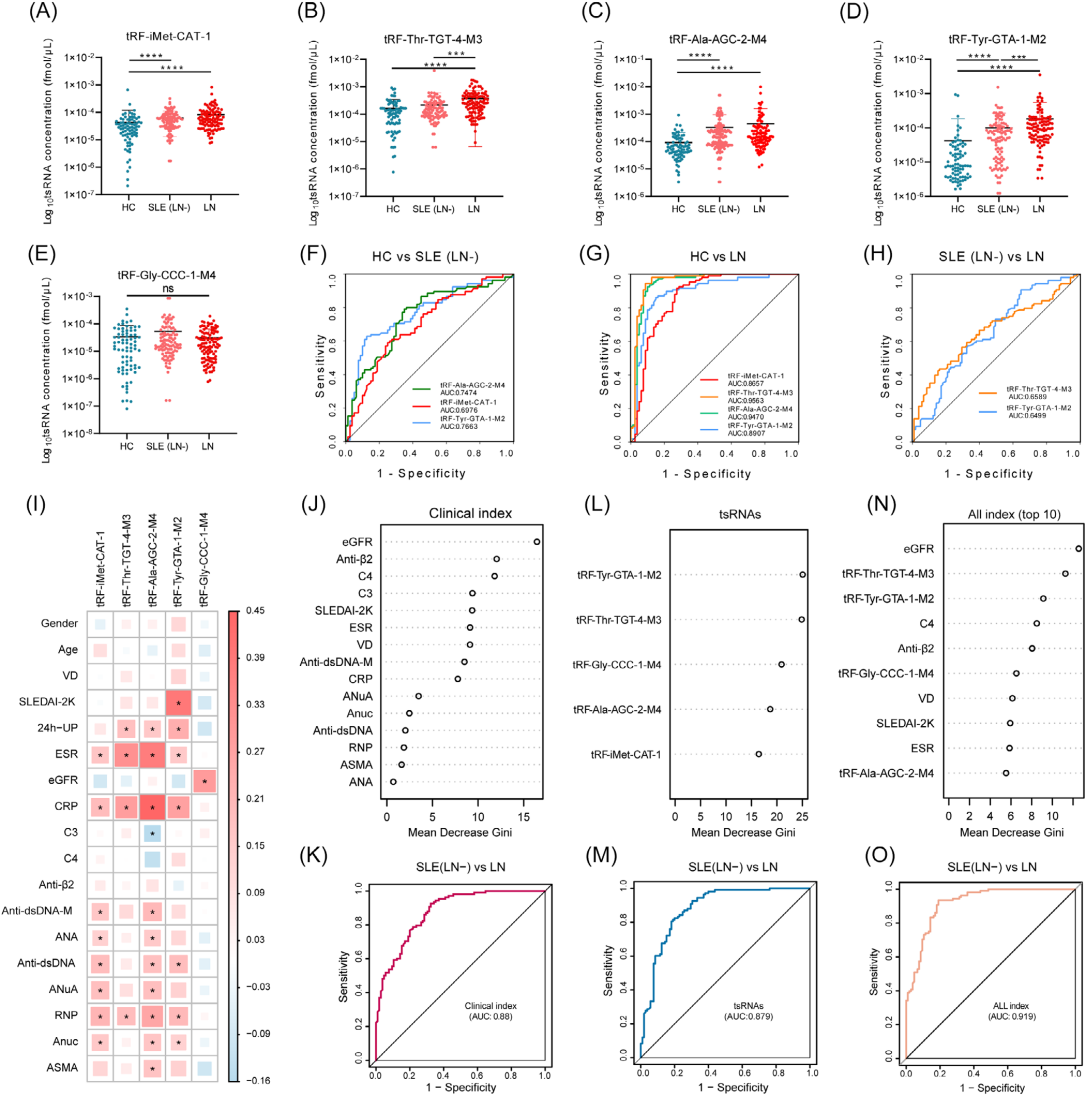
Clinical validation of extracellular vesicle‐encapsulated tsRNAs (EV‐tsRNAs) markers and construction of diagnostic models. (A‒E) Expression levels of tRF‐iMet‐CAT‐1, tRF‐Thr‐TGT‐4‐M3, tRF‐Ala‐AGC‐2‐M4, tRF‐Tyr‐GTA‐1‐M2 and tRF‐Gly‐CCC‐1‐M4 among the three groups of HC, systemic lupus erythematosus (SLE) (LN‒) and lupus nephritis (LN). (F) Receiver operating characteristic (ROC) curves of tRNA‐derived small non‐coding RNA (tsRNA) for the diagnosis of SLE (LN‒) and HC. (G) ROC curves of tsRNAs for the diagnosis of LN and HC. (H) ROC curve of tsRNAs for the diagnosis of SLE (LN‒) and LN. (I) Correlation heatmap between five tsRNAs and clinical detection indexes. (J) The mean decrease Gini of all clinical index diagnostic SLE (LN‒) and LN according to random forest analysis. (K) ROC curve of SLE (LN‒) and LN diagnosed by all clinical index predicted by random forest analysis. (L) The mean decrease Gini of five tsRNAs diagnostic SLE (LN‒) and LN according to random forest analysis. (M) ROC curve of SLE (LN‒) and LN diagnosed by five tsRNA predicted by random forest analysis. (N) Random forest analysis of tsRNAs and clinical indicators on the top 10 mean decrease Gini for SLE (LN‒) and LN diagnosis. (O) ROC curves of tsRNAs and clinical indexes for SLE (LN‒) and LN diagnosis by random forest analysis. Kruskal‒Wallis test was used to analyse the differences between groups. Spearman was used for correlation analysis (^*^ *p* < .05, ^***^ *p* < .001, ^****^ *p* < .0001).

Eventually, we predicted the structure and function of the screened tsRNAs using bioinformatics tools. tRF‐Thr‐TGT‐4‐M3 and tRF‐Tyr‐GTA‐1‐M2 were generated from tRNA‐Thr‐TGT‐4‐1 and tRNA‐Tyr‐GTA‐1‐1, respectively, both of which belong to the tRF‐3b type of tsRNA molecules (Figure 3A,D). Biological process, cellular component and molecular function showed that tRF‐Thr‐TGT‐4‐M3 was most significantly enriched in striated muscle cell development (GO: 0055002), striated muscle thin filament (GO: 0005865) and Tat protein binding (GO: 0030957) (Figure 3B). tRF‐Tyr‐GTA‐1‐M2 demonstrated enrichment in regulation of epithelial cell proliferation associated with prostate gland development (GO: 0060768), presynaptic active zone cytoplasmic component (GO: 0098831) and RNA polymerase II activating transcription factor binding (GO: 0001102) (Figure 3E). Interestingly, tRF‐Thr‐TGT‐4‐M3 was enriched in the vitamin D receptor pathway (WP2877), which is closely associated with the occurrence and development of SLE^10^ (Figure 3C). tRF‐Tyr‐GTA‐1‐M2 was enriched in bacterial invasion of epithelial cells (hsa05100) pathway, which may be a new explanation for bacterial infections in patients with LN (Figure 3F).

**FIGURE 3:**
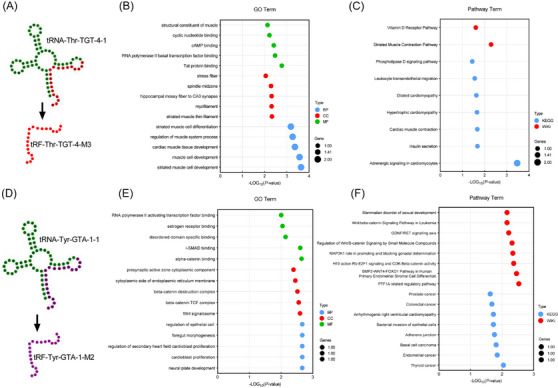
Biological structure and functional prediction of tRNA‐derived small non‐coding RNAs (tsRNAs). (A and D) Schematic of the biological structure of tRF‐Thr‐TGT‐4‐M3 and tRF‐Tyr‐GTA‐1‐M2. (B and E) GO enrichment analysis of tRF‐Thr‐TGT‐4‐M3 and tRF‐Tyr‐GTA‐1‐M2. (C and F) Pathway enrichment analysis of tRF‐Thr‐TGT‐4‐M3 and tRF‐Tyr‐GTA‐1‐M2.

Overall, we have identified several serum exosomal tsRNA biomarkers for diagnosing patients with SLE. Additionally, we have developed a diagnostic model that can distinguish the presence or absence of nephritis in SLE patients using combined tsRNAs. However, it is crucial to observe that this study is limited to a single‐centre sample, and subsequent validation is still required through multi‐centre, large‐scale investigations across different regions.

## AUTHOR CONTRIBUTIONS

*Methodology and data validation*: Ping Yang, Chenlan Wang and Yifan Sun. *Formal analysis*: Ping Yang, Zhiyang Li. *Guidance to the study*: Fangfang Jin, Zhiyang Li, Jin Wang and Yanbo Wang. *Methodology and writing—original draft*: Adeel Khan. *Writing—original draft*: Ping Yang and Zhiyang Li. *Collection of samples and clinical information*: Jianming Gong, Yiyuan Han. *Chief designer of the whole experiment*: Ping Yang. All authors have reviewed and approved the final manuscript.

## CONFLICT OF INTEREST STATEMENT

The authors declare they have no conflicts of interest.

## ETHICS STATEMENT

We adhered to the guidelines of the Declaration of Helsinki, and our research was endorsed by the ethics committee of Nanjing Drum Tower Hospital (ethics code: 2022‐466‐01, registration number of China Clinical Trial Registration Center: ChiCTR2100048082). Trial registration: Clinical Trial Registration Center, Registered 30 June 2021, https://www.chictr.org.cn/bin/home. Individuals whose faecal samples were part of the research willingly granted informed consent.

## CONSENT FOR PUBLICATION

Not applicable.

## Supporting information

Supporting Information

## Data Availability

The corresponding authors will be happy to share the datasets generated or analysed during the course of this investigation.
