# Supplementary material for: Serum exosomal tsRNA biomarkers: A novel strategy for identifying lupus nephritis
Source: Clin Transl Med. 2024 May 17;14(5):e1677. doi: 10.1002/ctm2.1677 (PMC11101668; doi:10.1002/ctm2.1677)
Supplement: Supplementary file 1 — Supporting Information [file CTM2-14-e1677-s001.docx]

**Supplementary materials**

**Materials and methods**

**Clinical sample collection**

All samples used to participate in the study were collected from September 2020 to June 2022 at Nanjing Drum Tower Hospital, affiliated with Nanjing University School of Medicine, and included 80 healthy subjects (HC), 122 SLE patients without nephritis (SLE(LN-)), and 131 LN patients. Samples were collected in accordance with the SLE classification criteria published by the Rheumatology Society in 1997, all subjects involved in this experiment gave written informed consent, and this study was approved by the Ethics Committee of Nanjing Drum Tower Hospital (ID: 2020-327-01). To obtain more accurate results, we applied to the order of sequencing, premerity screening (17 cases of SLE(LN-) and 23 cases of LN), verification (80 cases of HC, 105 cases of SLE(LN-) and 108 cases of LN) and clinical evaluation to start this experiment (**Table 1**)

**Isolation and extraction of serum-EVs**

Here, we isolated and extracted EVs from serum using the methods described in the literature. Briefly, we extracted serum Extracellular Vesicles (EVs) using the Total Exosome Isolation Kit (from serum) (Thermo Fisher Scientific, Inc., CN: 4478360) by taking fresh blood samples and letting them stand at 4°C for 2 h, centrifuging them at 3500 g for 10 min, taking the upper layer of serum and centrifuging it at 2000 g for 30 min. The supernatant was centrifuged at 10,000 g for 30 min, 100 μL of the serum supernatant was added with 20 μL of exosome isolation reagent, blown and mixed, and incubated on ice for 30 min. 10,000 g was centrifuged at room temperature for 10 min, and the resulting precipitates were EVs. 100 μL of PBS was used to resuspend the EVs, which were stored at -80°C or used immediately for subsequent experiments.

**Transmission electron microscopy (TEM)**

For electron microscopy, 20 μL of EVs sample was pipetted dropwise onto the copper grid and allowed to stand for 10 min at room temperature; 20 μL of 2% phosphotungstic acid was added dropwise onto the copper grid and negatively stained for 10 min, and excess dye was removed by blotting with filter paper; after drying of the copper grid, the EVs in the grid were photographed by TEM at 120 kV; The bilayer membrane structures were selected and the particles with diameters in the range of 100-200 nm were photographed and stored.

**Nanoparticle size tracking analysis (NTA)**

Polystyrene standard (PS) microspheres were configured at 1:250000 and PS microspheres were slowly injected into the NTA sample cuvette using a syringe. Autofocus the instrument, set the instrument sensitivity to 80 and wash the sample cuvette 3 times with purified water. Add 1.2 μL of EV sample to 1200 μL of purified water and mix by gentle inversion. Slowly inject the diluted sample into the sample cell with a syringe, check that the number of particles is around 200 per field, if not, wash the sample cell, load the reference diluted sample to the appropriate concentration from the previous time, inject again. With the sensitivity set to 80, enter the dilution factor, select the detection channel in EV mode and run the instrument; based on the instrument feedback results, the correct field of view data will be selected to generate EV detection reports.

**Western blotting (WB)**

EVs were lysed with RIPA lysis buffer containing protease inhibitors for 30 min on ice, centrifuged at 12000g for 10 min and the supernatant was collected. The protein concentration in the supernatant was quantified using a BCA protein assay kit. For denatured protein samples, 1/5 of the supernatant volume was added to 5 × SDS, boiled at 99°C for 5 min. Following the manufacturer's recommended protocol, 20 μg of protein was taken and loaded onto the concentrate, run at 80 V for 30 min and 120 V for 1 h on the separating gel. At a constant flow of 300 mA for 70 min, the proteins were transferred to a 0.2 µm PVDF membrane. The membranes were blocked with 5% skim milk for 1 h at room temperature, stripped, incubated with primary antibodies for CD63 (sc-5275, MX-49.129.5, Santa, USA), CD9 (sc-13118, C-4, Santa, USA), Alix (sc-53538, 3A9, Santa, USA) and Calnexin (sc-23954, AF18, Santa, USA) overnight at 4°C; the membrane was washed 4 times in 1 × TBST for 15 min, the membranes were incubated with the corresponding secondary antibodies for 1 h at room temperature, the membrane was washed 3 times in TBST for 10 min. Finally, ECL Exposure Liquid was added dropwise to the film, which was photographed and stored in the imaging system (**Figure S1**).

**Isolation and extraction of EV-RNAs**

Add 1 mL of Trizol lysate to the isolated EVs, vortex vertically for 30 s and leave for 5 min at room temperature; add 200 μL of chloroform to the solution, vortex for 15 s to a pink colour and leave for 7 min at room temperature; centrifuge at 16,000 g for 20 min at 4 °C, carefully aspirate 600 μL of supernatant and place in a new 1.5 mL EP tube; add 600 μL of isopropanol to the tube and mix by inverting up and down 10 times. After precipitation, centrifuge at 16,000 g for 20 min at 4 °C, carefully remove all liquid from the tube, add pre-cooled 75% ethanol and mix upside down; immediately centrifuge at 16,000 g for 20 min at 4 °C, carefully remove all liquid, invert onto absorbent paper and allow to dry for 5 min. Add 20 μL of DEPC treated water to the bottom of the tube and use immediately or store at -80 °C.

**Small RNAs sequencing**

Prior to sequencing, RNA samples were checked for integrity and concentration using agarose gel electrophoresis and Qubit; 3'-aminoacyl deacylation to 3'-hydroxyl was used for 3' adapter ligation and 3'-cyclic phosphate was moved to 3'-hydroxyl for phosphorylation to 5'-phosphate for 5'-adapter ligation, enzymatic removal of m1A, m1G and m3C methylation for efficient reverse transcription; selection of RNA biotype sequencing automated gel cutter Sequencing libraries were constructed, pass-checked and absolute quantified using an Agilent Bioanalyzer 2100; standard small RNA sequencing was performed using an Illumina NextSeq instrument, sequencing type 50 bp single-end reads; cytoplasmic tRNA sequences were downloaded from GtRNAdb and mitochondrial tRNA sequences were predicted using tRNAscanSE software. Mature tRNA library: predicted intron sequences removed and 3 terminal 'CCAs' added to each tRNA. Precursor tRNA library: the original tRNA sequence contains 40 nucleotides on either side. Data synthesis and statistical analysis was performed using the ArraystartRF&tiRNA-seq data analysis package.

**Real time fluorescence quantitative PCR (RT-qPCR)**

Primers for the tsRNAs were designed using miRNA Design V1.01, and all primers were synthesized in Nanjing Kingsray Biologicals. The cDNA was synthesized by Reverse Transcription PCR (RT-PCR) using miRNA 1st Strand cDNA Synthesis Kit (by stem-loop, Nanjing Novozymes Biologicals, China). After cDNA synthesis, RT-qPCR reactions were performed using the miRNA Universal SYBR qPCR Master Mix Kit to detect the expression of target tsRNAs, and a standard curve was used for absolute quantification of target tsRNA levels (**Figure S2**).

**Bioinformatics analysis**

Random forest model analysis: random forest training and prediction was performed using Python, and this study included 105 cases in the SLE (LN-) group and 108 cases in the LN group, reflecting a balanced distribution between the two groups. To adhere to widely accepted ML practices and mitigate potential biases, validation sets were utilized for hyperparameter optimization. The size of the validation set was determined as 30% of the corresponding training set size. Structural prediction of tsRNAs: Use the tRNAdb (http://trna.bioinf.unileipzig.de/DataOutput/Search) online software to find and download the Fast format of tsRNA secondary structure and map the secondary structure of tsRNAs. Functional prediction of tsRNAs: Use the tRFTar (http://www.rnanut.net/tRFTar/) online tool to predict the target genes of tsRNAs and perform pathway analysis such as GO and KEGG.

**Data statistics and analysis**

SPSS 24.0 and Graphpad prism 8.0 software were used for statistical analysis. Categorical data were expressed as frequencies and percentages (n, %), and the chi-square test was used for comparison between groups. Quantitative data from normal distribution were expressed as mean ± standard deviation (‾x ± SD) and t-test was used for comparison between groups. Quantitative data from non-normal distributions are expressed as M (P25, P75) and comparisons between groups were made using the Mann-Whitney U test.

**Figure legends**

**Figure 1** Development and screening of markers for EV-tsRNAs in lupus nephritis. (A) Volcano plot of differential expression of tsRNAs between SLE (LN-) and LN (Threshold setting: the fold change >2 and the P-value < 0.01); (B) Species distribution of tsRNAs in SLE (LN-) and LN; (C) Venn distribution of tsRNAs; (D) Length distribution of tsRNAs in SLE (LN-) and LN. (E) Expression Heatmap of top 10 high / low expression tsRNAs in LN compared to SLE (LN-) group. (F) Clinical sample validation of 10 candidate tsRNAs between SLE (LN-) and LN. Non parametric U-test was used to analyze the differences between the two groups. (^*^*P* < 0.05, ^**^*P*<0.01, ^***^*P*<0.001)

**Figure 2** Clinical validation of EV-tsRNAs markers and construction of diagnostic models. (A-E) Expression levels of tRF-iMet-CAT-1, tRF-Thr-TGT-4-M3, tRF-Ala-AGC-2-M4, tRF-Tyr-GTA-1-M2 and tRF-Gly-CCC-1-M4 among the three groups of HC, SLE (LN-), and LN. (F) ROC curves of tsRNA for the diagnosis of SLE (LN-) and HC; (G) ROC curves of tsRNAs for the diagnosis of LN and HC. (H) ROC curve of tsRNAs for the diagnosis of SLE (LN-) and LN. (I) Correlation heat map between five tsRNAs and clinical detection indexes. (J) The Mean Decrease Gini of all clinical index diagnostic SLE (LN-) and LN in random forest analysis. (K) ROC curve of SLE (LN-) and LN diagnosed by all clinical index predicted by random forest. (L) The Mean Decrease Gini of five tsRNAs diagnostic SLE (LN-) and LN in random forest analysis. (M) ROC curve of SLE (LN-) and LN diagnosed by five tsRNA predicted by random forest. (N) Random forest analysis of tsRNAs and clinical indicators on the top 10 Mean Decrease Gini for SLE (LN-) and LN diagnosis. (O) ROC curves of tsRNAs and clinical indexes for SLE (LN-) and LN diagnosis by random forest analysis. Kruskal Wallis test was used to analyze the differences between groups. Spearman was used for correlation analysis (^*^*P*<0.05, ^***^*P*<0.001, ^****^*P*<0.0001).

**Figure 3** biological structure and function prediction of tsRNAs. (A, D) Schematic of the biological structure of tRF-Thr-TGT-4-M3 and tRF-Tyr-GTA-1-M2; (B, E) GO enrichment analysis of tRF-Thr-TGT-4-M3 and tRF-Tyr-GTA-1-M2; (C, F) Pathway enrichment analysis of tRF-Thr-TGT-4-M3 and tRF-Tyr-GTA-1-M2.

**Supplementary Figure**


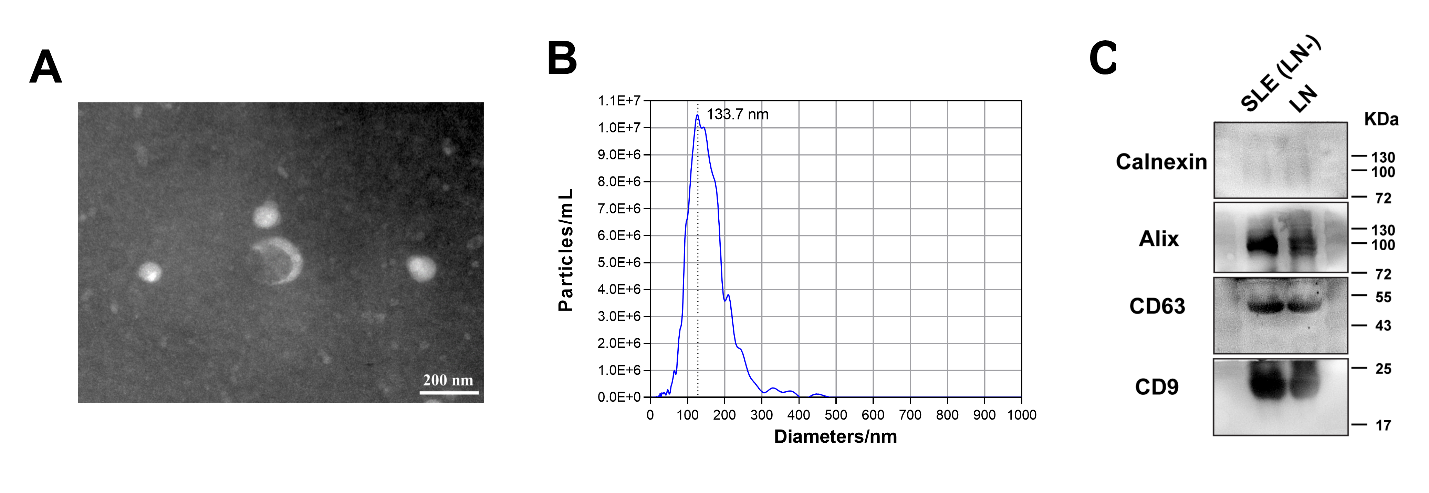


**Figure S1**: Characterisation of serum exosomes in patients with lupus nephritis. (A) Morphology of EVs under transmission electron microscopy (TEM); (B) Nanoparticle size tracking analysis the size distribution pattern of EVs (NTA); (C) Western blotting of EV specific membrane proteins (WB).


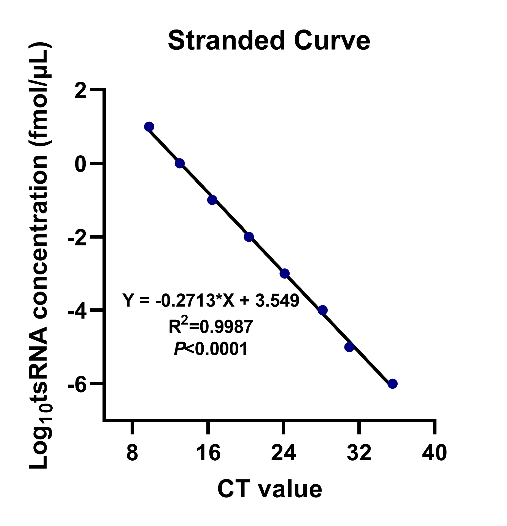


**Figure S2**: Standard curve between CT values of PCR and tsRNA concentration.


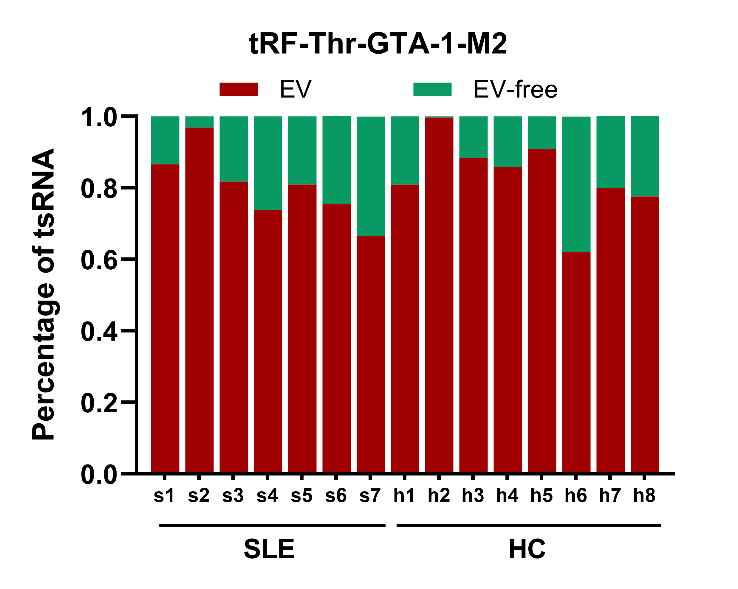


**Figure S3**: Percentage of tsRNA content in serum EVs and EV-free components.
